# Soundscapes influence the settlement of the common Caribbean coral *Porites astreoides* irrespective of light conditions

**DOI:** 10.1098/rsos.181358

**Published:** 2018-12-12

**Authors:** Ashlee Lillis, Amy Apprill, Justin J. Suca, Cynthia Becker, Joel K. Llopiz, T. Aran Mooney

**Affiliations:** 1 Woods Hole Oceanographic Institution, 266 Woods Hole Road, Woods Hole, MA 02543, USA; 2 MIT-WHOI Joint Program in Oceanography, 360 Woods Hole Road, Woods Hole, MA 02543, USA

**Keywords:** coral, acoustics, soundscape, larvae, settlement

## Abstract

The settlement of reef-building corals is critical to the survival and recovery of reefs. Recent evidence indicates that coral larvae orient towards reef sound, yet the components of the acoustic environment that may attract coral larvae and induce settlement are unknown. Here we investigated the effects of ambient soundscapes on settlement of *Porites astreoides* coral larvae using *in situ* chambers on reefs differing in habitat quality (coral and fish abundance). Mean larval settlement was twice as high in an acoustic environment with high levels of low-frequency sounds, typical of a high-quality, healthy reef; this result was observed in both natural light and dark treatments. Overall, the enhancement of coral settlement by soundscapes typical of healthy reefs suggests a positive feedback where soundscape properties of reefs with elevated coral and fish abundance may facilitate coral recruitment.

## 1. Introduction

Distributions of bottom-dwelling marine organisms are not random, and, in part, this is driven by differences in the supply and settlement of reproductive propagules (i.e. larvae). Most marine invertebrates with sessile juvenile and adult life stages, including reef-building corals, produce planktonic larvae whose settlement into a favourable habitat is critical to the maintenance and growth of adult populations. For these organisms, larval habitat selection and settlement are key life-history processes [1]. Settlement of planktonic coral larvae onto a suitable habitat is a determinant of future survival in corals and the biodiverse reef habitats they support [1,2]. Despite their small size and biological simplicity relative to many fish, crustacean and mollusc larvae (e.g. single larval development stage, lack of organs, no specialized swimming appendages), coral larvae display a range of behavioural and settlement responses to a combination of habitat-related physical, chemical and biological variables [3]. While poor swimming ability precludes the capacity to navigate horizontally to reefs against currents, detection of and responses to habitat cues confers some degree of active habitat selection by larvae at settlement, and this sensory capability increases the probability of successful coral recruitment [3,4].

Coral planulae respond to physical variables such as light and pressure, possibly facilitating their encounter with the substrate and the selection of favourable depths and light regimes in which to settle [5–7]. Reef water- and substrate-borne chemical cues, produced by resident organisms (e.g. algae, microbes, conspecifics), as well as local substrate properties such as texture and colour, are also known to influence coral larval settlement rates [5,8,9]. However, experiments investigating particular settlement cues often occur in laboratory environments, with cues in isolation, and not necessarily at ecologically relevant levels of stimuli. A major deficit in understanding the environmental drivers of settlement is translating the effects of a single cue to the ambient environment where larvae respond to complex cuescapes [3].

Biological and physical acoustic cues emanating from potential settlement sites can provide larvae with relevant sensory information complementary to the local substrate and water properties. Soundscape variation has been found to influence reef-fish and crustacean settlement [10–12], but its role in coral settlement is less understood. An initial behavioural study carried out in Curaçao showed that larvae of the reef-building coral *Orbicella faveolata* moved toward speakers playing reef sounds [13]. A field experiment using the same species and location examined coral settlement in relation to three reef soundscapes and revealed increased coral settlement under exposure to higher levels (5–10 dB) of low-frequency (25–1000 Hz) reef sounds [14], but light level variation at the sites was not controlled for and may have also impacted settlement patterns. Further, reef community characteristics, such as the benthic cover and fish assemblages, were not concomitantly quantified, thus limiting the interpretation of relationships between reef quality, soundscapes and settlement. Overall, these two initial works provide evidence that coral larvae can respond to reef soundscape elements, but relating the salient reef sounds to local reef characteristics and controlling for other environmental cues is needed to better understand the role of acoustic cues in coral settlement. This study investigated the effects of ambient (i.e. natural) soundscapes on the settlement responses of *Porites astreoides* coral larvae at well-characterized reef sites while testing for the light dependence of the acoustic cues. The aim was to expand upon the previous study of soundscape effects on coral settlement, using larvae of a common coral species in the Caribbean [15] and reefs with established links between soundscapes and community characteristics.

## 2. Material and methods

### 2.1. Coral collections, spawning and larval chambers

Eight colonies of the brooding coral *Porites astreoides* were collected on reefs off the island of St John, in the US Virgin Islands (18.31384° N, 64.76439° W) on 22 June 2017 from 10 m depth. These broodstock colonies were not collected at the experimental test sites but rather an adjacent reef outside the Virgin Islands National Park where we were permitted for coral collection. Prior to the field tests, colonies were maintained in a shaded outdoor ambient seawater-supplied aquarium. Six coral colonies spawned overnight during the July new moon (22–24 July) and larvae were collected each morning and maintained in 0.2 µm filtered seawater. Larval production ranged from 53 to 388 larvae over three nights. On 25 July, larvae from all colonies and spawning nights were pooled, and 18 groups of 55 actively swimming larvae were selected (990 total larvae). Groups were randomly assigned to one of nine light or nine dark 140 ml polypropylene chambers (preconditioned with reef water for one month) filled with 0.7 µm filtered seawater to remove zooplankton grazers but retain seawater bacteria and smaller phytoplankton.

Each chamber contained two preconditioned (one month) settlement surfaces: a clay stilt (3.8 cm diameter) and a red cable tie (10.2 cm length; shown in an earlier study [16] to attract settling coral larvae). While these surfaces do not represent naturalistic substrate, such as coral rubble or crustose coralline algae, for coral larvae to encounter, they were selected to provide standardized substrate and surface area across treatments and to limit the influence of other (i.e. chemical) cues. Light chambers were transparent, allowing ambient light ingress, while dark chambers were identical to light chambers except externally covered with black tape to prevent light penetration. Absolute light levels and quality were not measured during experiments, as the aim here was not to create specific light treatments or investigate effects of light on the settlement, but rather to control for any between-site light differences and test whether the presence or absence of light would influence responses to potential acoustic cues.

### 2.2. Settlement experiments

**Figure 1.**
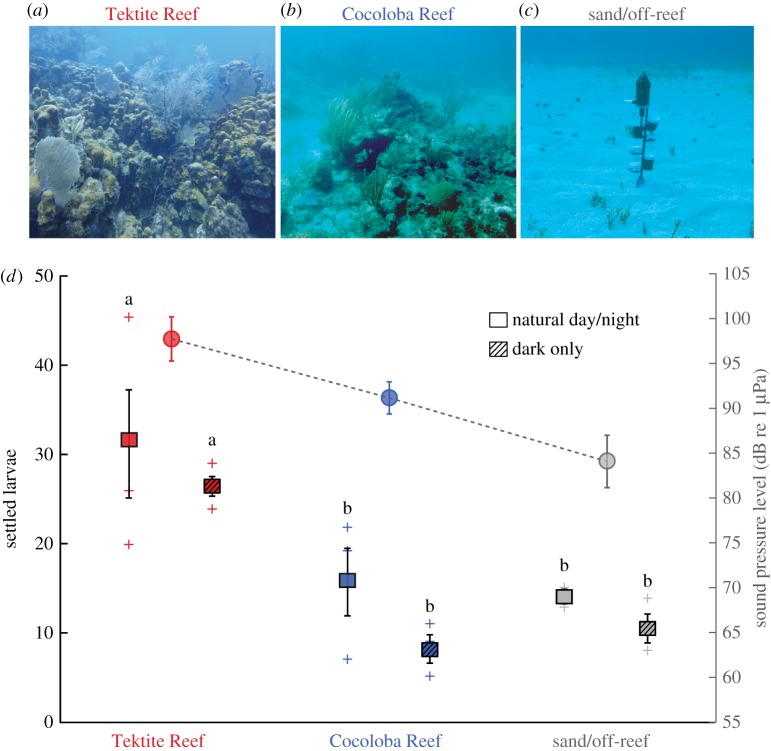
Larval settlement varied according to site and sound pressure level. Photographs of study sites (*a*) Tektite Reef, (*b*) Cocoloba Reef and (*c*) off-reef sand with experimental set-up. (*d*) Squares represent mean (±s.d.) larval settlement in the light and dark chambers (left axis), with symbol plus (+) indicating settlement values for each replicate. Settlement was significantly higher at Tektite compared with the other sites, in both the dark and light treatments (two-way ANOVA, *p* < 0.01, ‘a’ versus ‘b’). Circles indicate mean sound pressure levels (SPL ± s.d.) in the low-frequency band (right axis) during the experiment, with the line signifying the SPL trend.

Following larval addition, three light and three dark settlement chambers were each affixed to a 0.75 m vertical pole deployed at three sites: Tektite Reef (18.30962° N, 64.72218° W), Cocoloba Reef (18.31528° N, 64.76065° W) and an off-reef sand site with no reef structure within 100 m (18.31789° N, 64.75059° W) (table 1 and figure 1*c*). Sites differed in biophysical habitat characteristics (table 1) known to influence soundscape properties [17]. The experimental set-up included acoustic recorders (SoundTrap ST-300, Ocean Instruments NZ), recording continuously at 48 kHz, and temperature/light loggers (HOBO Pendant UA-002, Onset Corporation). A recorder and HOBO logger were affixed at the top of each experimental pole, approximately 0.75 m above the seafloor. The chambers and instruments were secured 0.2–0.5 m above the seafloor in 7–10 m water depth (figure 1*c*). Light sensors were positioned facing upward and sampled every 10 min. The six chambers on each pole were positioned in random vertical order, alternating the side of the pole to which they were affixed so that no chamber was directly above another, to limit shading effects. For the reef sites, the experimental set-up was placed in the approximate centre of the reef structure.

**Table 1.** Description of study sites, including benthic coverage, fish abundance and ambient light environment recorded during the experiments.

	characteristic	Tektite Reef	Cocoloba Reef	off-reef sand
benthic coverage^b^ (mean ± s.d. %)	hard coral	27.0 ± 5.2	6.5 ± 4.5	0
	soft coral	1.0 ± 1.1	5.2 ± 2.1	0
	sponge	13.7 ± 1.0	0.5 ± 0.5	0
	macroalga	41.2 ± 1.1	55.8 ± 10.6	12.3 ± 6.0
	cyanobacterial mats	5.7 ± 5.5	0.5 ± 1.2	0
	sand	2.7 ± 2.4	28.0 ± 13.0	87.7 ± 6.0
	rubble	7.5 ± 14.2	1.3 ± 1.8	0
	other^a^	1.3 ± 1.0	2.2 ± 2.2	0
fish^c^	mean abundance (±s.d.)	165.7 ± 76.2	51.0 ± 16.9	10.0 ± 6.6
	species richness	36	21	2
ambient daytime light	lux (mean ± s.d.)	1170 ± 872	1525 ± 1253	7340 ± 6753

^a^Other category includes hydroids, dead coral and pavement.

^b^Six benthic survey transects were conducted at each site.

^c^Three fish video transects were conducted at each site.

Larvae were completely isolated within settlement chambers, allowing exposure to ambient sounds (polypropylene plastics have high acoustic transparency with attenuation between 0.24 and 0.5 dB mm^−1^ [18]) while preventing exposure to other water-borne habitat cues (e.g. reef water chemicals). Chambers were recovered after 62 h and maintained in seawater tables during the 6-h processing period in which settled corals were enumerated. The use of static filtered seawater was necessary to isolate putative acoustic cues from water-borne cues. While this may present concerns about coral larval health, previous coral larval rearing and laboratory experiments have used static culturing techniques with far higher densities over similar time periods without water quality or mortality issues [13,14]. In our study, unsettled actively swimming larvae were still present, suggesting that conditions remained sufficient for larval survival. We did not detect dead larvae or particulate matter from decaying larvae in the chambers, further indicating that chamber conditions were suitable for the survival of the larvae.

### 2.3. Soundscape and habitat characterization

Acoustic recordings were analysed to compare the experimental soundscapes, initially by examining acoustic spectra (sound power as a function of frequency). Digital recording samples were analysed using MATLAB code written to compare the acoustic characteristics of the three sites for the duration of the experiment. Frequency composition of the ambient soundscapes was compared using acoustic spectra (sound power as a function of frequency) across measured frequencies (50–20 000 Hz). Mean power spectral densities were estimated (Hamming window, non-overlapping 0.5-s windows) within 1-min samples across the total experiment length (62 h). Root-mean-square (RMS) sound pressure levels (SPL; dB re 1 µPa) were calculated for each site in the 1-min samples, within two frequency bands of interest. The lower analysis band (50–1000 Hz) contains the majority of fish-produced acoustic signals, as well as noise generated by wind and waves, while the higher analysis band (1000–20 000 Hz) primarily represents the acoustic energy derived from invertebrate sounds (e.g. snapping shrimp) [17]. Additionally, to assess differences in acoustic variables between study sites in more detail, values of RMS octave-band levels (dB re 1 µPa; centroid frequencies at 62.5, 125, 250, 500, 1000, 2000, 4000, 8000 and 16 000 Hz) were generated for each 1-minute sample and a Kruskal–Wallis test was applied to test the effect of experimental site on octave-band levels. Because reef soundscapes exhibit diel variability, spectrograms were produced to further visually characterize acoustic differences between sites.

Benthic cover and fish diversity and abundance at the sites were characterized during July 2017 using SCUBA-based visual surveys as previously described [17]. Benthic surveys included six 10 m-long transects, with benthic cover recorded every 10 cm. For fish, three 30 m-long, 2 m-wide video transects were performed at each site, with fish enumerated and identified. Fish abundances were calculated as the total number per transect, and fish species richness was the total number of species identified at each site. Fish were infrequent and in low abundance at the off-reef sand site, and thus the occasional fish was counted, identified by the diver and recorded underwater.

## 3. Results

### 3.1. Larval settlement greatest on reef with abundant fish and coral

Coral larvae settled in all chambers, primarily on clay stilts (5–43 chamber^−1^) with minimal settling on red zip ties or chamber walls (0–4 chamber^−1^). Larval settlement differed significantly by site (two-way ANOVA, *F*_2,12_ = 11.021, *p* < 0.01; table 2) and was significantly greater for chambers at Tektite Reef compared with Cocoloba Reef and the off-reef sand site, irrespective of the light environment (Tukey's pairwise comparisons, difference of means = 14.0–16, *p* < 0.05; table 2). Tektite Reef also had the highest coral and fish abundance and diversity (table 1), compared with Cocoloba Reef and the off-reef sand site (figure 1). The elevated settlement at Tektite Reef compared to the other sites was approximately two times higher in the natural light treatment and two to three times higher in the dark treatment (figure 1*d*). Notably, the standard deviations were generally smaller in the dark than light treatments for all sites.

**Table 2.** Summary of coral settlement two-way ANOVA statistical comparisons and Tukey's pairwise comparisons. (d.f., degrees of freedom; sum Sq, sum of squares; Dm, difference of means).

test	d.f.	sum Sq	*F*-value	*p*-value
reef	2	880	11.0	0.002*
light/dark	1	80	2.0	0.182
pairwise comparisons				

comparison	Dm	*p*	*q*	*p*-value
Tektite versus Cocoloba, light and dark	15.0	3	5.82	0.004*
Tektite versus off-reef sand, light and dark	14.7	3	5.68	0.005*
Off-reef sand versus Cocoloba, light and dark	0.33	3	0.13	0.996
Tektite versus off-reef sand, light only	15.3	3	4.20	0.029*
Tektite versus Cocoloba, light only	14.0	3	3.84	0.046*
Cocoloba versus off-reef sand, light only	1.33	3	0.37	0.964
Tektite versus Cocoloba, dark only	16.0	3	4.39	0.023*
Tektite versus off-reef sand, dark only	14.0	3	3.84	0.046*
Off-reef sand versus Cocoloba, dark only	2.0	3	0.55	0.921

*p < 0.05.

### 3.2. Soundscape differed between sites during experiment

**Figure 2.**
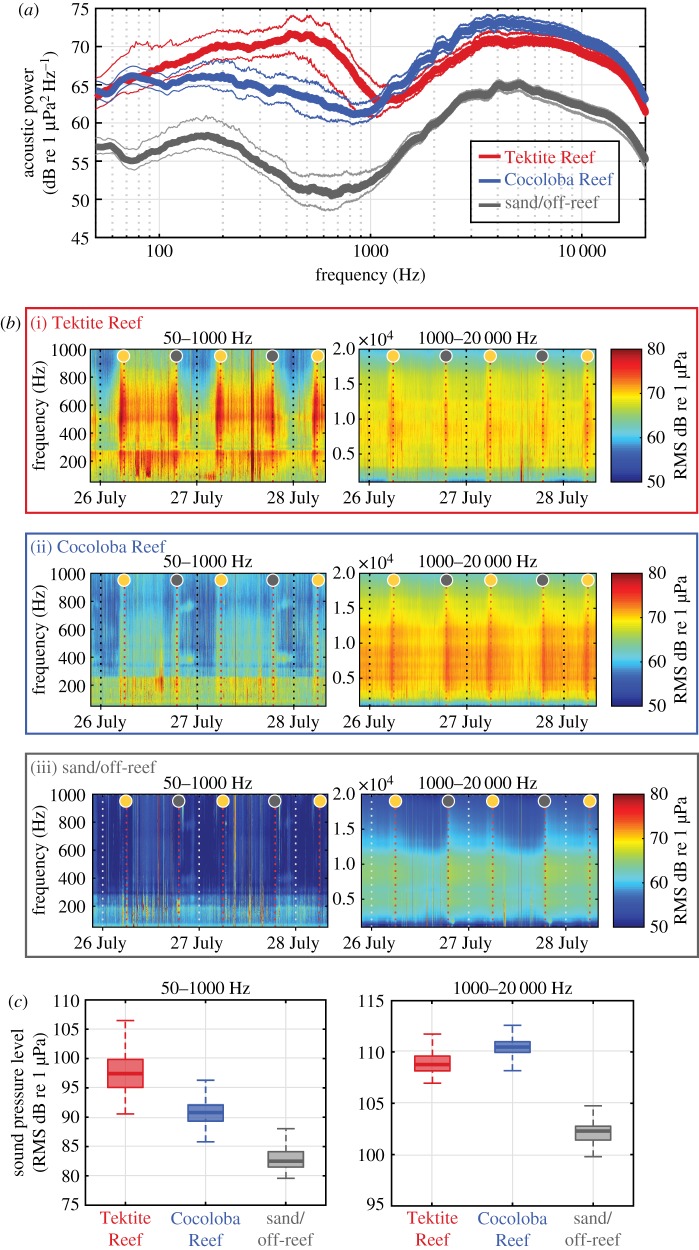
Inter-site variation in soundscape characteristics during settlement experiments. (*a*) Acoustic power spectra (dark lines denote median, light lines, interquartile range) showing different spectral shapes at Tektite Reef compared to Cocoloba Reef and the off-reef site (average PSD estimated via Welch's method, 1.5 Hz frequency resolution, 0.5 s windows). (*b*) Spectrograms for low-frequency (left panel) and high-frequency (right panel) bands at each site illustrate strong diel low-frequency fish chorusing at Tektite (i), high-frequency snapping-shrimp crepuscular peaks at Cocoloba (ii) and the off-reef sand site (iii). Grey and yellow circles indicate sunset and sunrise, respectively. (*c*) Boxplots (median values as solid horizontal lines, 50th percentile values as box outline and 90th percentile values as whiskers) of SPL summarize overall differences between sites in the sound levels present.

Sound levels differed significantly between experimental sites across frequencies (figure 2*a*, octave-band analysis: Kruskal–Wallis, *p* < 0.001). Tektite Reef was highest in lower-frequency acoustic amplitudes. Low frequencies on coral reefs tend to reflect higher fish abundance and diversity and are also the frequencies sensitive to many marine invertebrates [17,19,20]. Higher frequency sounds, indicative of snapping shrimp, were greatest at Cocoloba Reef (figure 2*d*). The Tektite Reef soundscape differed from the other sites predominantly with a notable peak in acoustic power between 300 and 800 Hz (figure 2*a*). Cocoloba Reef and the off-reef site showed a similar frequency composition (figure 2*a*), with broad peaks in spectra from 100 to 300 Hz and 2 to 10 kHz, but Cocoloba Reef was approximately 10 dB (*ca* 3×) louder.

Temporal trends reveal marked acoustic differences among sites (figure 2*b*). In the lower-frequency, fish-dominated band, Tektite Reef demonstrated crepuscular chorusing, with increased activity throughout the daytime (figure 2*b*(i)); this fish chorusing was less evident at Cocoloba Reef and absent off-reef (figure 2*b*(ii,iii)). In the high-frequency snapping-shrimp band, Cocoloba Reef and the off-reef site showed strong crepuscular peaks and night-time dominance while Tektite Reef exhibited asymmetrical peaks (figure 2*b*). Overall, reef soundscapes were higher in sound levels at all frequencies (figure 2*c*) compared with the off-reef site, and Tektite Reef showed the highest sound levels in the fish-dominant frequency band.

## 4. Discussion

This field experiment was designed to test the effect of habitat-related soundscape variability on the larval settlement of a common Caribbean brooding coral *Porites astreoides*. Our data establish that soundscape variation can influence coral settlement in this species, regardless of light environment, and demonstrate highest larval settlement under exposure to the soundscape of a healthier reef environment, with abundant hard corals, sponges and fishes. Previously, larvae of the free-spawning coral *Orbicella faveolata* were found to orient and move towards reef sound [13] and to show a settlement response corresponding to reef soundscape levels in a low-frequency band (25–1000 Hz) [14]. Similarly, the enhanced larval settlement at Tektite Reef appears most likely related to the soundscape differences present in lower frequencies which typically include many fish calls [21]. Indeed, fish abundance and diversity were higher at Tektite Reef. Interestingly, a graded settlement response to the SPL in the low-frequency band (50–1000 Hz) was not detected, i.e. Cocoloba Reef did not generate settlement levels between those observed at Tektite Reef and the off-reef site. Still, Cocoloba Reef and the off-reef site soundscapes differed primarily in sound intensity rather than any other obvious spectral or temporal qualities (e.g. same spectral shapes, figure 2*a*). This implies that the acoustic driver of larval response to the Tektite Reef soundscape was more specific than simply higher sound levels, and could have resulted from the sounds of specific fish (e.g. the 300–800 Hz peak) and/or the strong crepuscular chorusing that were both absent from the Cocoloba Reef and off-reef sites. An alternative possibility is that coral larvae may only respond to low-frequency (50–1000 Hz) acoustic cues above a certain intensity threshold. This would also lead to the results we observed if the threshold intensity fell between that of Tektite Reef and Cocoloba Reef. Distinguishing between the two possible drivers of the observed results (fish calls or intensity threshold) warrants further study.

Together, the habitat surveys, acoustic monitoring and settlement results indicate a possible link between habitat quality, soundscape and coral settlement whereby diverse and abundant soniferous communities may provide cues for settling corals. However, precisely which acoustic cues are important and whether variation in their levels or diel patterns affect settlement remain open questions. Moreover, while this study expands knowledge of coral settlement processes by establishing that soundscape cues can influence settlement for a coral species with a brooding reproductive strategy, the roles of genetic variation, parental effects and local adaptation in this larval response are unknown. Further work is needed to test how widely applicable acoustic settlement cues are across coral species and geographies.

Coral larval settlement was enhanced at Tektite Reef both in the dark and ambient light treatments, suggesting that the soundscape is a cue used both in the natural light and dark environments. While settlement responses to soundscape variation were found irrespective of light environment, the influence of soundscape was most pronounced when coral larvae were required to settle in the dark. Sound may be a particularly important cue in light-limited environments such as at night or on mesophotic reefs and is a compelling avenue for future settlement cue investigation. Owing to the nature of the light scattering environment, our study sites did differ in light intensity, but this variation did not appear to relate to observed settlement patterns. There also tended to be greater standard deviations under natural light compared with dark conditions. Perhaps this was related to the naturally varying light cue, or perhaps there is an interaction of light and sound conditions. Further study would be needed to disentangle the individual and synergistic effects of these variables. To our knowledge, no previous studies have examined coral settlement in darkness, and here we demonstrate that the settlement of algal symbiont-bearing corals can indeed occur in darkness. Coral larvae are known to have settlement preference for particular light intensities [22] and spectral qualities [7], and future multifactorial experiments are needed to establish the relative influences of light and acoustic properties on coral settlement. In addition, experiments to identify diel patterns of coral settlement (i.e. does settlement occur continuously or preferentially at night or day?) are essential to inform hypotheses about which soundscape elements may be important and how these cues interact with the light regime.

Overall, we demonstrate that increased coral settlement occurs in the presence of a low-frequency, fish-chorus dominated soundscape. Such a soundscape is often representative of a healthier, more diverse reef. Further, the influence of the soundscape is important to coral larvae independent of light environment. The coral larvae in this study appear to respond to the soundscape of a favourable settlement site, shown here to be a reef with higher sound levels in fish calling acoustic frequencies. This suggests a positive feedback loop where reefs of higher coral cover and fish abundance generate soundscapes best suited to attract coral larvae, which in turn sustain the healthy coral community and habitat for fish (similar to a mechanism proposed for fish [23]). It follows that reefs in poor health could struggle to attract the coral recruits needed for recovery. Yet, with further work to establish the relevant cues, playback treatments of low-frequency reef-fish communities could be employed in restoration schemes. While relationships between reef quality and soundscape have been previously documented [17,24], this study provides new details on the relationship between reef habitat and biota, the soundscape, light environment and coral settlement. Given that reefs face increasing anthropogenic pollution, including noise [25] and soundscape alteration through habitat degradation [23], understanding the role of sound-mediated settlement in coral recruitment may be critical to enhancing coral populations and conserving reefs.

## Data accessibility

Data are available online (https://www.bco-dmo.org/project/659919).

## References

[1] Harrington L, Fabricius K, De'ath G, Negri A 2004 Recognition and selection of settlement substrata determine post-settlement survival in corals. Ecology 85, 3428– 3437. (10.1890/04-0298)

[2] Babcock R, Mundy C 1996 Consequences of settlement choice for early growth and survivorship in two scleractinians. J. Exp. Mar. Biol. Ecol. 206, 179–201. (10.1016/S0022-0981(96)02622-6)

[3] Gleason D, Hofmann D 2011 From gametes to recruits. J. Exp. Mar. Biol. Ecol. 408, 42–57. (10.1016/j.jembe.2011.07.025)

[4] Butman C, Grassle J, Webb C 1988 Substrate choices made by marine larvae settling in still water and in a flume flow. Nature 333, 771–773. (10.1038/333771a0)

[5] Gleason D, Edmunds P, Gates R 2006 Ultraviolet radiation effects on the behavior and recruitment of larvae from the reef coral *Porites astreoides*. Mar. Biol. 148, 503–512. (10.1007/s00227-005-0098-y)

[6] Stake J, Sammarco P 2003 Effects of pressure on swimming behavior in planula larvae of the coral *Porites astreoides* (Cnidaria, Scleractinia). J. Exp. Mar. Biol. Ecol. 288, 181–201. (10.1016/S0022-0981(03)00018-2)

[7] Mundy C, Babcock R 1998 Role of light intensity and spectral quality in coral settlement: implications for depth-dependent settlement? J. Exp. Mar. Biol. Ecol. 223, 235–255. (10.1016/S0022-0981(97)00167-6)

[8] Dixson D, Abrego D, Hay M 2014 Chemically mediated behavior of recruiting corals and fishes: a tipping point that may limit reef recovery. Science 345, 892–897. (10.1126/science.1255057) 25146281 PMC4470392

[9] Gleason D, Danilowicz B, Nolan C 2009 Reef waters stimulate substratum exploration in planulae from brooding Caribbean corals. Coral Reefs 28, 549–554. (10.1007/s00338-009-0480-1)

[10] Stanley J, Radford C, Jeffs A 2010 Induction of settlement in crab megalopae by ambient underwater reef sound. Behav. Ecol. 21, 113–120. (10.1093/beheco/arp159)

[11] Simpson S, Meekan M, McCauley R, Jeffs A 2004 Attraction of settlement-stage coral reef fishes to reef noise. Mar. Ecol. Prog. Ser. 276, 263–268. (10.3354/meps276263)

[12] Radford C, Jeffs A, Montgomery J 2007 Directional swimming behavior by five species of crab postlarvae in response to reef sound. Bull. Mar. Sci. 80, 369–378.

[13] Vermeij MJ, Marhaver KL, Huijbers CM, Nagelkerken I, Simpson SD 2010 Coral larvae move toward reef sounds. PLoS ONE 5, e10660 (10.1371/journal.pone.0010660) 20498831 PMC2871043

[14] Lillis A, Bohnenstiehl D, Peters JW, Eggleston D 2016 Variation in habitat soundscape characteristics influences settlement of a reef-building coral. PeerJ 4, e2557 (10.7717/peerj.2557) 27761342 PMC5068343

[15] Green DH, Edmunds PJ 2008 Increasing relative abundance of *Porites astreoides* on Caribbean reefs mediated by an overall decline in coral cover. Mar. Ecol. Prog. Ser. 359, 1–10. (10.3354/meps07454)

[16] Mason B, Beard M, Miller M 2011 Coral larvae settle at a higher frequency on red surfaces. Coral Reefs 30, 667–676. (10.1007/s00338-011-0739-1)

[17] Kaplan MB, Mooney TA, Partan J, Solow AR 2015 Coral reef species assemblages are associated with ambient soundscapes. Mar. Ecol. Prog. Ser. 533, 93–107. (10.3354/meps11382)

[18] Selfridge AR 1985 Approximate material properties in isotropic materials. IEEE Trans. Son. Ultrason. 32, 381–394. (10.1109/T-SU.1985.31608)

[19] Packard A, Karlsen HE, Sand O 1990 Low frequency hearing in cephalopods. J. Comp. Physiol. A 166, 501–505. (10.1007/BF00192020)

[20] Mooney TA, Hanlon RT, Christensen-Dalsgaard J, Madsen PT, Ketten DR, Nachtigall PE 2010 Sound detection by the longfin squid (*Loligo pealeii*) studied with auditory evoked potentials: sensitivity to low-frequency particle motion and not pressure. J. Exp. Biol. 213, 3748–3759. (10.1242/jeb.048348) 20952625

[21] Lobel PS, Kaatz IM, Rice AN 2010 Acoustical behavior of coral reef fishes. In Reproduction and sexuality in marine fishes: patterns and processes (ed. Cole KS), pp. 307–386. Berkeley, CA: University of California Press.

[22] Maida M, Coll J, Sammarco P 1994 Shedding new light on scleractinian coral recruitment. J. Exp. Mar. Biol. Ecol. 180, 189–202. (10.1016/0022-0981(94)90066-3)

[23] Gordon TA, Harding HR, Wong KE, Merchant ND, Meekan MG, McCormick MI, Radford AN, Simpson SD 2018 Habitat degradation negatively affects auditory settlement behavior of coral reef fishes. PNAS 115, 5193–5198. (10.1073/pnas.1719291115) 29712839 PMC5960293

[24] Lammers M, Zang E, Kaplan MB, Mooney TA, Fisher-Pool PI, Brainard R 2017 Variation in the soundscapes of Pacific coral reefs over multiple spectral, temporal, and spatial scales. J. Acoust. Soc. Am. 142, 2502 (10.1121/1.5014138)

[25] Kaplan MB, Mooney TA 2015 Ambient noise and temporal patterns of boat activity in the US Virgin Islands National Park. Mar. Poll. Bull. 98, 221–228. (10.1016/j.marpolbul.2015.06.047) 26254882

